# Reflections on the implementation of an acute general surgical COVID‐19 roster at North Shore Hospital, Auckland – a prospective observational study

**DOI:** 10.1111/ans.19402

**Published:** 2025-03-05

**Authors:** Jamie‐Lee Rahiri, Rebecca Teague, Teresa Holm, Jason Tuhoe, Jonathan Koea

**Affiliations:** ^1^ Department of General Practice and Primary Healthcare The University of Auckland Auckland New Zealand; ^2^ Te Piringa Kōtuku Tuhauora Medical Associates Auckland New Zealand; ^3^ North Shore Hospital Te Whatu Ora Waitematā Auckland New Zealand; ^4^ Auckland Hospital Te Whatu Ora Te Toka Tūmai Auckland New Zealand; ^5^ Department of Surgery The University of Auckland Auckland New Zealand

**Keywords:** COVID‐19, general surgery, pandemic, workforce

## Abstract

**Background:**

Nearly 5 years after the arrival of coronavirus disease (COVID‐19) in New Zealand (NZ), many lessons have been learned. At North Shore Hospital (NSH) in Auckland, NZ, a general surgical COVID‐19 Crisis Roster (CCR) was established for the first lockdown in 2020. This study summarizes the prospective monitoring of our CCR and offers a framework for adapting our roster for future pandemics.

**Methods:**

A prospective observational review of all acute general surgical admissions (from 30 March 2020 to 26 April 2020) was performed and compared with admissions over the same period in 2019.

**Results:**

A total of 443 patients were admitted to NSH during the CCR period compared with 552 patients in 2019 (−19.8%, *P* = 0.001). The rate of acute cholecystectomies increased (+54.5%, *P* = 0.002) whilst operations related to carbuncle/cyst excision (−83.3%, *P* < 0.02), endoscopy (−62.5%, *P* = 0.04), and surgical interventions for postoperative complications (−72.2%, *P* = 0.03) decreased. No significant differences in the rate of (re)admissions for postoperative complications or grade of complication were observed (*P* = 0.66). Within the context of no surgical team members contracting COVID‐19, the cancellation of outpatient clinics, and elective operating lists, the CCR was deemed feasible and easy to implement.

**Conclusion:**

While patient safety was not compromised during the implementation of our pandemic roster, we advocate that our roster should be adapted and improved to include Māori health expertise, a prospective monitoring data expert committee and our nursing and allied health staff should we seek to use this CCR in future.

## Graphical abstract

The implementation of our pandemic roster was feasible and did not compromise patient care in the first lockdown in New Zealand. However, we advocate that our roster should be adapted and improved to include Māori health expertise, a prospective monitoring data expert committee and our nursing and allied health staff should we seek to re‐use this CCR in future.

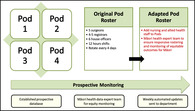

## Introduction

The arrival of coronavirus disease (COVID‐19) to New Zealand (NZ) in early 2020 saw a swift change to our healthcare system. Dubbed as ‘unprecedented times’, the necessity to move quickly to prepare for overwhelming surges in COVID‐19 cases while continuing to provide acute surgical care was paramount.^1^ As described previously, the NZ government instituted a four‐level alert system with specific public health and social measures to mitigate further community spread.^2^ On 25 March 2020, NZ moved to *Alert Level 4*, where strict measures were imposed, including the closure of all non‐essential services and businesses and mandatory self‐isolation of all New Zealanders.

**Fig. 1:**
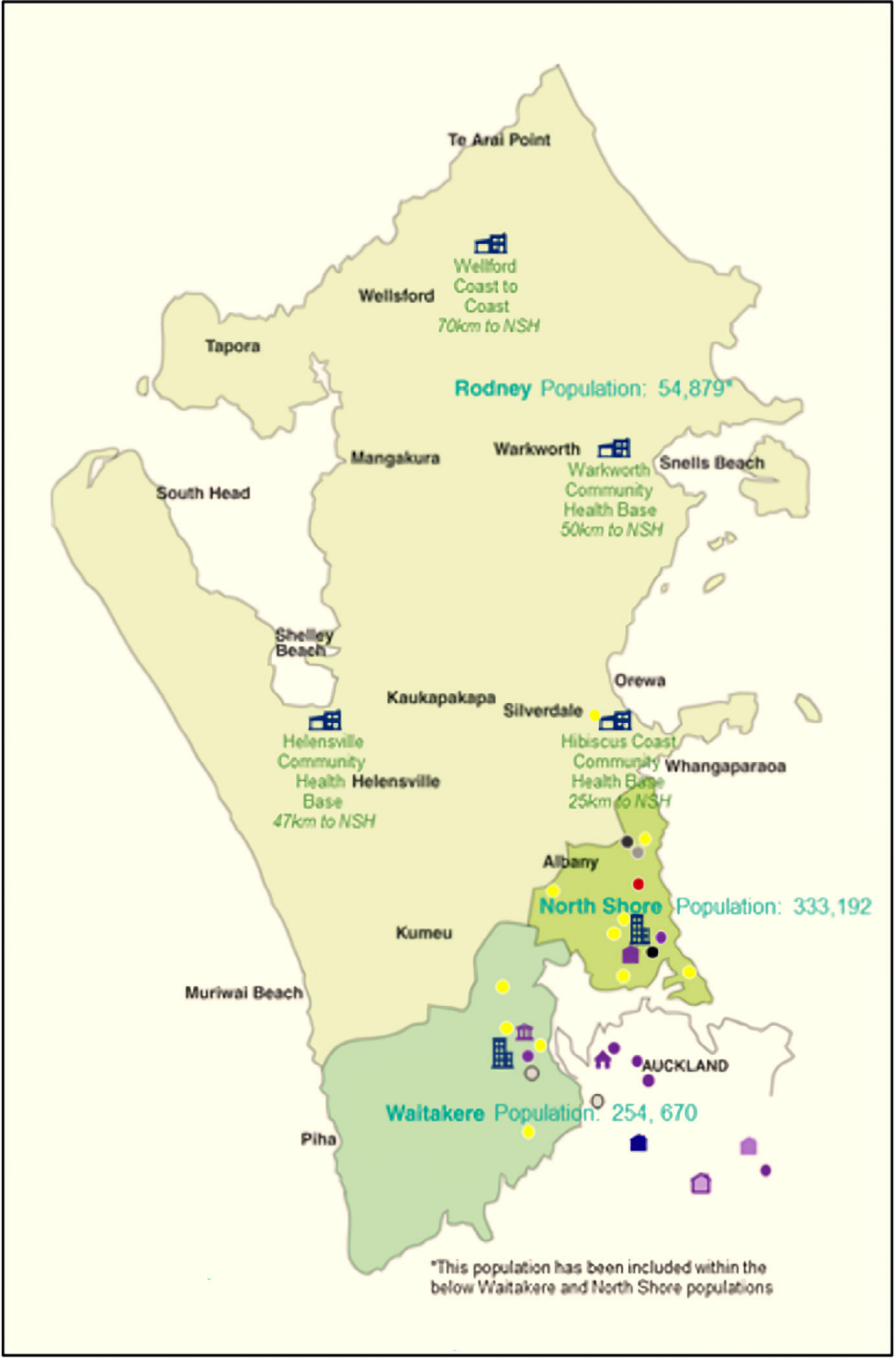
Population profile of Te Whatu Ora Waitematā adapted from Te Whatu Ora Waitematā^4^

In 2021, six metropolitan general surgical departments in NZ described how they adapted new rosters and restructured teams, of which our centre was one.^3^ North Shore Hospital (NSH) provides secondary and tertiary level care to the Northern and Western regions of Auckland, NZ, serving a population of more than 630 000, the largest and one of the most rapidly growing regions in NZ (Fig. 1).^4^ At the start of the first lockdown, NSH coalesced seven surgical teams into a four‐pod COVID‐19 crisis roster (CCR) and all elective surgical lists and clinics (excluding cancer cases) were postponed (Table 1).^5^

**Table 1:** Comparison of the new Waitematā DHB COVID‐19 crisis and traditional rosters^5^

COVID‐19 roster	Traditional roster
Four large pods made up of 4 consultant surgeons, 4–5 registrars and 5–6 house officers.	Seven surgical teams consisting of 2–3 consultant surgeons, 2 registrars and 2–3 house officers.
Rotating of 4 days of 12‐h shifts per pod	Acute patients admitted under an individual consultant surgeon who is on call for 24 h
Staff remain in their pods and do restrict physical contact with members of other pods.	Acute admissions led by a junior registrar who holds an acute admitting phone daily (0730–2230)
Consultant surgeons hold the acute admitting phone daily (0730–1930)	

The NZ government's national COVID‐19 elimination strategy in 2020 was highly successful, notably due to the collective and holistic approaches of Māori, who sought to protect their families and communities.^6^ Given the historical context of Māori suffering disproportionately in previous pandemics, there were concerns about potential high morbidity and mortality rates during COVID‐19.^7^ With this knowledge, Māori communities exercised self‐determination (tino rangatiratanga) by establishing pandemic coordination hubs on marae, implementing roadblocks, and providing local testing and vaccination efforts.^8^, ^9^, ^10^ The impact of the NZ COVID‐19 pandemic management on Māori health inequities at both a nationwide and regional level is currently being assessed.^11^ When equity and Te Tiriti o Waitangi are not considered in the design and implementation of services or emergency rosters, we rely on the retrospective application of these frameworks, which is not ideal.

The first case of COVID‐19 in NZ was identified on February 28th, 2020. The primary goal of the surgical department at NSH was to treat all acute and elective patients as safely and efficiently as possible while minimizing surgical workforce ‘loss’ due to COVID‐19. In preparation for the commencement of the CCR on March 30th, a prospective database was established on 27 March 2020, to monitor patient flow and care through our department. This prospective observational study followed all acute general surgical admissions and operations over the CCR period at NSH compared to those over the same period in 2019 as a way to evaluate the safety and feasibility of the CCR at NSH during the first government‐mandated COVID‐19 national lockdown in NZ. Additionally, based on our experience with this pandemic roster, we constructed an adaptive framework and strategic plan to improve this CCR for future use.

## Material and methods

A prospective observational cohort study of all patients admitted to the general surgical department at NSH, Auckland, NZ, during the CCR period, from 30 March to 27 April 2020, was performed. Patients were prospectively followed from admission to discharge, and clinical information relevant to their care was collated into a prospective database. Comparative data pertaining to all acute surgical admissions over the same period in 2019 were extracted retrospectively from the electronic clinical records. Two reviewers made comparisons between both periods (J‐LR, RT). This study is reported in line with the Strengthening the Reporting of Observational Studies in Epidemiology Guidelines.^12^

### Data collection

The National Health Index numbers of all patients admitted to the NSH Department of Surgery were prospectively retrieved and entered into our database using the General Surgery Audit Tool on Clinical Portal, the patient electronic medical management software utilized at NSH. The prospective database was constructed using Microsoft Excel 2018 and required manual oversight by two study authors (J‐LR and RT). Once data extraction was complete, all data was de‐identified and retrospectively analyzed and reported. The same data variables were extracted from admission to discharge for both the CCR period and the comparator period in 2019:

1. Patient demographics (age, gender, ethnicity, comorbidities, NZDep18 scores): NZDep18 is an area‐based measure of socioeconomic deprivation in New Zealand. It measures the level of deprivation for people in each small area and is based on nine NZ census variables.^13^
2. Diagnosis
3. Time to diagnostic interventions (imaging modality) and operative intervention
4. Post‐operative complications using the Clavien‐Dindo classification^14^
5. Hospital length of stay and readmissions

### Statistical analysis

Patients were grouped by the year of admission, diagnosis, and procedure for statistical analysis. In this study, we knew we would not achieve equal explanatory power for Māori, so we did not compare outcomes by ethnicity. Not achieving equal explanatory power in ethnicity studies can lead to significant methodological biases, statistical limitations, and potential misrepresentation of Indigenous health experiences.^15^ All continuous variables are presented as mean (SD) unless stated. Unless stated otherwise, descriptive results are produced as counts with proportions for categorical variables and mean with standard deviation for continuous variables. Statistical analysis was performed using IBM SPSS Statistics Version 29.0.2.0 (Chicago, Il, USA). Chi‐square and Mann–Whitney U tests were used for univariate analyses of categorical and non‐parametric continuous variables, respectively, with values of <0.05 considered statistically significant.

### SARS‐CoV‐2 testing during the CCR

The commercial diagnostic rapid antigen tests available today for SARS‐CoV‐2 testing were absent in early 2020 in NZ.^16^ During the CCR, testing for SARS‐CoV‐2 was done so using polymerase chain reaction testing where the turnaround time for results was at least 60 minutes. This meant that testing had to be selective, that is, for those patients with a high suspicion of exposure or those undergoing procedures that would be difficult to control for viral transmission, such as endoscopic procedures.

### Māori responsiveness

Our study was led and governed by Māori surgical academics (JL‐R and JK) and ensured Māori oversight and mentorship in building Māori surgical scholarship capacity. Furthermore, this research aligns with a structural determinants approach critiquing the implementation of our CCR surgical roster and providing reflections on how this could (and should have) been designed to be more responsive to Māori in our catchment area.^17^ This research rejects racism, opposes deficit theory and advocates for social justice and health equity for Māori by retrieving through the retrieval of this research space in surgery.^18^

### Ethical approvals

All research procedures complied with relevant laws and institutional guidelines per the ethical approvals granted by the Human Disability and Ethics (Reference 20/NTB/74) and the Waitematā District Health Board Ethics Committees.

## Results

### Demographics

Over the CCR study period (30 March to 27 April 2020), 447 admissions were recorded compared to 552 over the same period in 2019. Most patients identified as NZ European ethnicity, were primarily female, predominantly aged between 50 and 79, and lived in areas of low deprivation (Table 2). The mean overall number of patient admissions over the four‐week CCR period was 111 ± 15 (SD).

**Table 2:** Patient demographics for the CCR period (Marcy–April 2020)

	March–April 2020	March–April 2019	*P*‐value
Total admissions	443	552	0.001*
Age (years + range)	52.2 (15–98)	50.3 (15–99)	0.08
Length of stay (days) mean ± SD	2.7 ± 3.8	3.1 ± 4.5	<0.01*
Gender			
Female	249		0.07
Male	191		
**Ethnicity**			
NZ European	247	280	0.009*
Other European	73	84	
Asian	44	76	
NZ Māori	42	48	
Pacific†	29	40	
Other‡	8	24	
**Deprivation (NZDep18 score)**			
1–2	43	60	0.005*
3–4	165	173	
5–6	144	188	
7–8	74	103	
9–10	16	26	
N/A overseas resident	1	7	
**Charlson comorbidity index**			
0	170	271	0.004*
1	82	64	
2	63	73	
3	49	64	
4	46	58	
5§	33	22	
**Admission pods**			
1	118		
2	116	N/A	N/A
3	124		
4	85		

* Statistically significant *p* < 0.05.

† Samoan, Tongan, Cook Islander, Tokelauan.

‡ Middle Eastern, African, Latin American/Hispanic.

§ Severity of comorbid diseases: mild, CCI scores of 1–2; moderate, CCI scores of 3–4; and severe, CCI scores ≥5.

### Admitting diagnoses

**Fig. 2:**
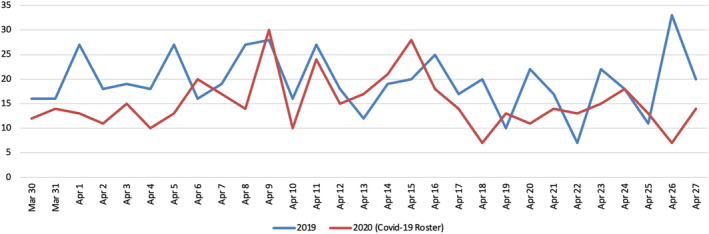
Comparison of admissions between lockdown in 2020 to the same period in 2019.

Table 3 shows that, on average, there was a mean decline in daily admissions by 19.8% during the CCR period (*P* < 0.001). Figure 2 displays the daily number of admissions over both periods. Overall, no significant differences were observed for each diagnosis group besides patients admitted with gastroenteritis (−58.8%, c^2^ 1, *P* < 0.01). However, there was a significant reduction in the number of patients admitted with choledocholithiasis (−80%, c^2^ 1, *P* < 0.02) alongside a significant increase in patients admitted with symptomatic cholelithiasis (+200%, c^2^ 1, *P* < 0.001). Overall, the majority of trauma admissions for the CCR and 2019 periods were for blunt trauma injuries (*N* = 20 vs. *N* = 23, respectively, c^2^ 1, *P* < 0.57). Of these, the most common injuries were rib fractures with or without a pneumothorax (*N* = 10 vs. *N* = 11, c^2^ 1, *P* < 0.97).

**Table 3:** Comparison of acute admissions between March and April 2019 and 2020

	March–April 2020 (*N* = 443)	March–April 2019 (*N* = 552)	% change 2020 versus 2019	*P*‐value
Total admissions	443	552	−19.8%	0.001*
Appendicitis	29	36	−19.4	0.8
Biliary	78	79	−1.3	0.16
Cholecystitis	41	36	13.9	0.11
Pancreatitis	17	27	−37.0	0.42
Symptomatic cholelithiasis	14	0	+100%	0.10
Cholangitis	3	1	200.0	0.22
Choledocholithiasis	3	15	−80.0	0.02*
Cancer	16	14	14.3	0.3
Colitis	9	7	28.6	0.3
Diverticulitis	21	36	−41.7	0.2
Perforated	4	10	−60.0	
Gastritis	7	17	−58.8	0.1
Gastroenteritis	0	16	−100.0	<0.01*
Incarcerated hernia	7	12	−41.7	0.5
Intestinal	44	36	22.2	0.05
Obstruction	22	17	29.4	0.17
Perforation	2	1	100.0	0.44
Volvulus	7	3	133.3	0.10
Haemorrhage – lower	8	9	−11.1	0.83
Haemorrhage – upper	5	2	150.0	0.15
IBD	0	3	−100.0	0.12
Other	52	63	−9.5	0.73
Perianal disease**	6	9	−34.6	0.50
Postoperative complications	30	31	−3.2	0.45
Soft tissue infection	67	95	−28.4	0.43
Abscess*	46	52	−9.6%	
Cellulitis	0	7	−100.0	
Carbuncle	11	22	−50.0	
Mastitis	7	12	−41.7	
Other (ulcers)	3	2	50.0	
Trauma	23	25	−8.0	0.6
Unspecified abdominal pain	54	76	−28.9	0.5

* Includes perianal, pilonidal and all other sites.

** Includes haemorrhoids, fissure and fistula‐in‐ano. IBD, inflammatory bowel disease.

### Acute operations

Overall, fewer acute general surgical operations were performed during the CCR period than in 2019, although this finding was statistically insignificant (−22.6%, c^2^ 1, *P* = 0.63). However, during the CCR period, the rate of acute cholecystectomies increased by 54.5% (+54.5%, c^2^ 1, *P* = 0.002), and the number of procedures related to carbuncle/cyst excision, endoscopy, and surgical interventions for postoperative complications decreased significantly (Table 4).

**Table 4:** Comparison of acute operations between March and April 2019 and 2020

	March–April 2020 (*N* = 181)	March–April 2019 (*N* = 234)	% change 2020 versus 2019	*P*‐value
Appendicectomy	37	41	−9.8%	0.59
Open	0	2		
Cholecystectomy	51	33	+54.5%	0.002*
Laparoscopic	47	32		
Subtotal cholecystectomy	3	0		
Open	1	1		
Colectomy	1	1	0	0.88
Emergency laparotomy	15	17	−10%	0.79
Endoscopy	9	24	−62.5%	0.04*
Colonoscopy	0	9		
Flexible sigmoidoscopy	3	0		
Gastroscopy	6	15		
Examination of the rectum (GA)	3	9	−66.7%	0.17
Haemorrhoidectomy	0	1		
Excision of cyst/carbuncle	2	12	−83.3%	<0.02*
Hernia repair	7	8	−12.5%	0.78
Open mesh repair of ventral hernia	3	4		
Open inguinal hernia repair with mesh	2	3		
Open repair of femoral hernia	2	1		
Incision and drainage of abscess	44	56	−21.4%	0.91
Wound debridement/exploration	3	9	−66.7%	0.17
Postoperative intervention	5	18	−72.2%	0.03*
Other	3†	7‡	−57.1%	0.77

* Statistically significant *p* < 0.05.

### Post‐operative complications

Similar rates of admissions were recorded during the CCR period compared to 2019 (*N* = 30 vs. *N* = 31, −3.2%, c^2^ 1, *P* = 0.45). Despite this, a significantly lower rate of acute operations was performed to remediate surgical complications during the CCR period (−72.2%, c^2^ 1, *P* = 0.03). There were no significant differences in complication grades utilizing the Clavien‐Dindo classification system, including mortality (*N* = 2 for CCR vs. *N* = 3 in 2019, *P* = 0.66).^14^

### SARS‐CoV‐2 testing

A total of 144 patients met the threshold to be tested for SARS‐CoV‐2 over the CCR period, with seven patients initially assigned the status of ‘suspected COVID‐19’. However, no patients returned a positive test result, and of all patients tested for the virus, *N* = 63 (44%) proceeded for an operation. Lastly, no surgical team members contracted SARS‐CoV‐2 during the CCR period. As such, the general surgical service could complete the CCR pod system with a full surgical workforce.

## Discussion

**Fig. 3:**
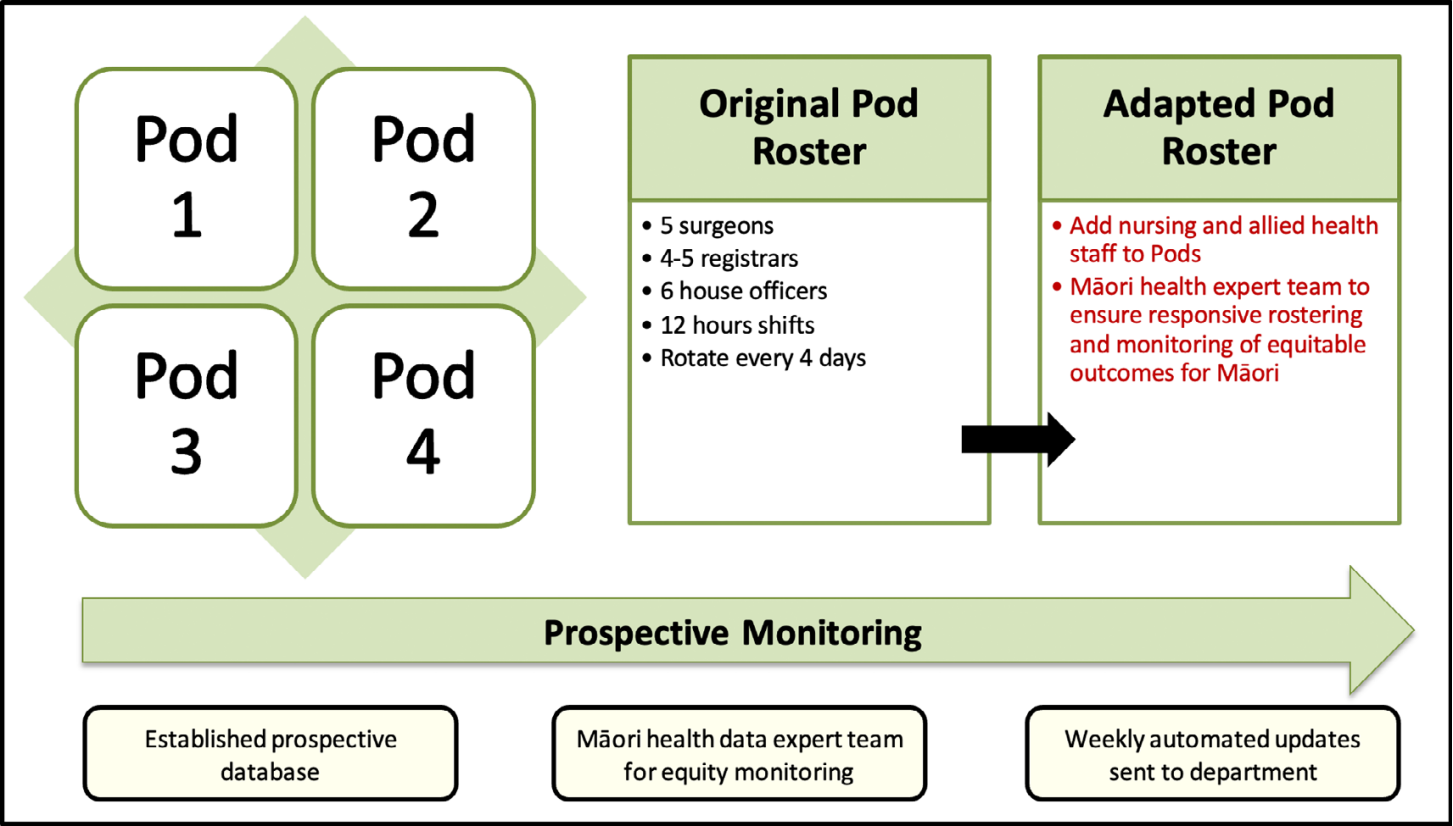
Adaptation framework and strategy for future implementation of the CCR.

This study showed a reduction in acute general surgical admissions and operations during the four‐week CCR within our first COVID‐19 lockdown in NZ. The rate of acute cholecystectomies increased significantly during this period, while acute operations for endoscopy, postoperative complications, and carbuncle/cyst excisions decreased compared to the same period in 2019. Importantly, there were no significant differences in the rates of admissions or acute surgical operations for postoperative complications. With less than 2 weeks to design and implement this CCR, we reflected on our experience and presented a framework and plan to further evolve our roster in the event of another pandemic (Fig. 3).^19^

During the initial wave of COVID‐19, New Zealand recorded low case numbers, infection rates, and fatalities compared to other high‐income countries, thanks to prompt national measures. Between 2 February and 13 May 2020, NZ recorded 1503 COVID‐19 cases, including 95 hospitalizations and 22 deaths.^20^ The fact that we had no COVID‐19 cases at NSH over the CCR period reflects the notably low rate of COVID‐19 community transmission in NZ at that time. Reduced acute surgical admissions due to lower rates of community transmission was a common trend within surgical units worldwide, particularly in the earlier stages, In Ireland, O'Connell *et al*.^21^ reported a 42% decrease in admissions to their unit from March to April 2020 compared with the same period in 2019.^21^ Similarly, in Canada, Balvardi *et al*.^22^ described a 27% decrease in admissions between March and May 2020 compared with the same period in 2019.^22^ A large study in the United States reviewed all paediatric and adult patients undergoing surgical procedures in 49 states during the initial 2020 COVID‐19‐related shutdown from March – to April 2020, compared with the same period in 2019.^23^ They reported a decrease in surgical procedure volume to nearly half of baseline rates, and upon reopening, the rates of surgical procedures rebounded to 2019 levels. These were maintained throughout the peak burden of patients with COVID‐19 beyond April 2020.^23^ This example shows that developed countries' health systems can return to pre‐pandemic surgical capacity with adequate resources, allowing surgical departments to adapt and deliver care during a pandemic.

Inspecting the diagnostic subpopulations of our study more closely, we found a significantly higher rate of acute cholecystectomies performed during the CCR period. Hessheimer *et al*.^24^ observed a slightly higher admissions rate for biliary pathology between February and May 2020 than in 2019.^24^ They suggested that this could be attributed to lifestyle behaviours impacted by COVID‐19 that are known to exacerbate biliary tract disease, such as exercising less, poor sleep, and binge eating.^24^, ^25^ However, we speculate that the significant surge in acute cholecystectomies during the CCR period was primarily attributable to the cancellation of our elective operative lists. This meant that as we were running an entirely acute general surgical department over the CCR period, all patients who required acute surgery could be completed as there were fewer surgical lists to compete with. It is essential to highlight that NSH usually services a large elective general surgical workload. Toh *et al*.^3^ compared COVID‐19 responses by surgical departments between the three Auckland hospitals, Waikato, Canterbury and Capital Coast Districts. They highlighted that NSH served the largest catchment population of 628 970 people, with the highest elective admissions at 4197 per annum, nearly twice that of most other hospitals.^3^

Reducing elective surgical resources and personnel while ensuring the safe delivery of acute surgical care during the COVID‐19 pandemic was the main goal in preparing for an influx of patients with COVID‐19. Surgical departments worldwide shifted resources to reduce access to elective surgical care, except surgical oncology.^26^ Additionally, telemedicine evolved rapidly and has become commonplace in surgical follow‐up clinics due to the economic benefits and mitigation of patient barriers, such as missing work and hospital travel.^27^, ^28^, ^29^ The initial responses by surgical departments to COVID‐19 occurred in a crisis. On reflection, many of the structural changes to surgical services, including surgical workforce rostering, were tried and tested on the run and evaluated retrospectively. The pod‐based staffing model from COVID‐19 hospital surges is effective for various crises, utilizing self‐contained, cross‐functional teams that operate autonomously with clear command structures. It can be applied in disaster response command centres, emergency operations facilities, field hospitals, and mobile medical units, benefiting organizations facing sudden service demand increases while maintaining quality standards.^3^

Upon the arrival of the COVID‐19 pandemic, a need for evidence‐based literature providing clinical and organizational guidelines for the management of general surgery departments, including rostering, was apparent. An outbreak of COVID‐19 in any surgical department would be disastrous, posing a significant risk to patients who require acute surgical care. Au *et al*.^30^ instituted a system similar to our CCR in their Emergency Department, with six teams rotating every 4 days with 12‐h daily shifts.^30^ The rotation of six teams was viable as it allowed for more extended rest periods, which would be beneficial if a team became incapacitated so a ‘resting’ team could cover for that team. Additionally, this roster included nursing staff, whereas our CCR only involved doctors (consultant surgeons, registrars and house officers). While pragmatic challenges were posed, Au *et al*.^30^ shared that their pandemic roster significantly improved team members' camaraderie, which is synonymous with our experience.^30^

Finally, the sole focus of this CCR was surgical workforce preservation and mitigation of COVID‐19 transmission among our patients. Therefore, the CCR did not consider Māori health equity. Māori health equity is a legislated right reaffirmed by multiple constitutional documents, namely, Te Tiriti o Waitangi.^31^, ^32^ While NZ achieved a lower COVID‐19 mortality than many countries, the NZ public health response was less effective for Māori than non‐Māori including the vaccination rollout strategy and the rapid withdrawal of the COVID‐19 protection measures before adequate vaccination coverage was met for Māori.^33^, ^34^ Approximately 10% of the total population served by NSH (630 000 people) identify as Māori, and in our study, 9.5% of patients admitted during the CCR were Māori. Despite this, we could not establish a Māori health data expert team to ensure robust processes for monitoring equity for Māori. Therefore, in our adaptation plan for the CCR roster, we have advocated for implementing a Māori health expert team to ensure responsive rostering and monitoring of equitable outcomes for Māori.

### Limitations

As a prospective observational study, we acknowledge that there are better study designs to assess the safety and feasibility of a new roster. Our CCR was designed and implemented within 2 weeks, which conferred a limited timeframe for developing a robust prospective database. We also recognize that transitioning to a primarily acute general surgical service allowed our clinical workforce to provide more focused care for patients in the ward and operating theatre due to cancelling all outpatient clinics and elective operating lists. Lastly, we were fortunate that no staff members at NSH contracted COVID‐19 through the CCR period. Therefore, we were not faced with the challenge of losing members of our workforce to COVID‐19, which would have more stringently tested the safety and feasibility of the CCR roster. We also acknowledge that utilizing patient complication rates and staff contraction rates of COVID‐19 are not the best test markers for determining roster feasibility; however, in the pandemic setting with limited planning time, roster planning becomes an urgent and complex challenge requiring rapid, flexible strategies as seen with our CCR. Given our increased understanding of surgery in a pandemic setting, future studies should incorporate more robust measures and could monitor changes in departmental fatigue and the relationship with patient outcomes.

## Conclusions

This study presents a single‐centre experience of the institution of a COVID‐19 pandemic general surgical roster, which showed a significant reduction in the number of daily admissions and acute operations in line with international trends. In addition, based on the measures employed in our study, patient safety was not compromised throughout the implementation of our pandemic roster. Given our experience with this roster, we would re‐use this in the event of another pandemic with adaptations to include health equity and Te Tiriti o Waitangi while also incorporating our other clinical and allied health staff.
